# Diagnosis of central serous chorioretinopathy by deep learning analysis of *en face* images of choroidal vasculature: A pilot study

**DOI:** 10.1371/journal.pone.0244469

**Published:** 2021-06-18

**Authors:** Yukihiro Aoyama, Ichiro Maruko, Taizo Kawano, Tatsuro Yokoyama, Yuki Ogawa, Ruka Maruko, Tomohiro Iida

**Affiliations:** Department of Ophthalmology, Tokyo Women’s Medical University, Shinjuku, Tokyo, Japan; Massachusetts Eye & Ear Infirmary, Harvard Medical School, UNITED STATES

## Abstract

**Purpose:**

To diagnose central serous chorioretinopathy (CSC) by deep learning (DL) analyses of *en face* images of the choroidal vasculature obtained by optical coherence tomography (OCT) and to analyze the regions of interest for the DL from heatmaps.

**Methods:**

One-hundred eyes were studied; 53 eyes with CSC and 47 normal eyes. Volume scans of 12×12 mm square were obtained at the same time as the OCT angiographic (OCTA) scans (Plex Elite 9000 Swept-Source OCT^®^, Zeiss). High-quality *en face* images of the choroidal vasculature of the segmentation slab of one-half of the subfoveal choroidal thickness were created for the analyses. The 100 *en face* images were divided into 80 for training and 20 for validation. Thus, we divided it into five groups of 20 eyes each, trained the remaining 80 eyes in each group, and then calculated the correct answer rate for each group by validation with 20 eyes. The Neural Network Console (NNC) developed by Sony and the Keras-Tensorflow backend developed by Google were used as the software for the classification with 16 layers of convolutional neural networks. The active region of the heatmap based on the feature quantity extracted by DL was also evaluated as the percentages with gradient-weighted class activation mapping implemented in Keras.

**Results:**

The mean accuracy rate of the validation was 95% for NNC and 88% for Keras. This difference was not significant (*P* >0.1). The mean active region in the heatmap image was 12.5% in CSC eyes which was significantly lower than the 79.8% in normal eyes (*P*<0.01).

**Conclusions:**

CSC can be automatically diagnosed by DL with high accuracy from *en face* images of the choroidal vasculature with different programs, convolutional layer structures, and small data sets. Heatmap analyses showed that the DL focused on the area occupied by the choroidal vessels and their uniformity. We conclude that DL can help in the diagnosis of CSC.

## Introduction

Artificial intelligence (AI) or machine learning using deep learning (DL) techniques has achieved human-like or even beyond human performances especially in visual recognition. In the Go and Shogi board games, AI is comparable to or has surpassed top-level human players, and it has led to the creation of new moves that humans could not imagine [1–3]. Despite such remarkable achievements, one of the problems of AI or DL is its lack of transparency. Because the contents of the DL are in a black box, it is difficult to completely explain even by human experts the reasons used by DL for the choices made even which raises concerns about accountability and responsibility.

In the field of ophthalmology, AI has been applied for the diagnosis and/or staging of diabetic retinopathy [4–6], glaucoma [7–9], age-related macular degeneration [10, 11], retinopathy of prematurity [12–14], and other retinochoroidal disorders including central serous chorioretinopathy (CSC) [15–20]. Most of the AI methods enabled ophthalmologists to diagnose and determine the stage of the disorders with the same or slightly higher accuracy than specialists. However, where to find and produce the results are some of the undetermined factors.

CSC is a chorioretinal disorder that is associated with a serous retinal detachment in the macular region including the fovea which then leads to visual impairments [21]. The primary change is in the choroid where there is a choroidal thickening and a dilation of the large choroidal blood vessels in the middle or Haller’s layer [22–27]. We have reported that the choroidal vascular density in one-half of the choroid is higher in eyes with CSC than normal eyes using *en face* OCT images [28]. However, it was difficult to diagnose CSC based on only the choroidal vascular density.

The purpose of this study was to determine whether the choroidal vascular pattern in the OCTA *en face* images is different in CSC eyes from that of normal eyes using DL. In addition, we determined the regions of the choroidal vasculature that were used by DL to make the differentiations.

## Methods

The medical record of 100 eyes of 100 patients who had been examined by OCT and OCTA in the Department of Ophthalmology of the Tokyo Women’s Medical University between 2017 and 2018 were analyzed. The procedures used were approved the Institutional Review Board of the Tokyo Women’s Medical University School of Medicine, and they conformed to the tenets of the Declaration of Helsinki (approval number 2636-R). All of the examinations were performed after an informed consent was obtained from the patients. In our department, OCT and OCTA devices are used routinely to study eyes with macular and retinal disorders, and observational studies of CSC and age-related macular degeneration.

Fifty-three eyes of 49 patients (39 men and 10 women) with CSC were studied. Their average age was 51.7 ± 12.7 years, and all had been diagnosed with CSC by fluorescein angiography (FA) and indocyanine green angiography (ICGA). Eyes treated with photodynamic therapy were excluded because of choroidal changes after photodynamic therapy [29–32]. Forty-seven eyes of 47 individuals (11 men, 36 women; average age 37.5 ± 5.4 years) without retinal and choroidal disorders were examined in the same way, and their data were compared to those of the CSC patients.

### Obtaining OCTA and OCT *en face* images

All eyes were examined by a swept source OCTA device (SS-OCTA; AngioPlex Elite 9000, Zeiss, Germany) whose light source emission was between 1040 and 1060 nm. The SS-OCTA cannot obtain images of the choroidal blood flow pattern in normal eyes because of the attenuation of the observation light by the RPE. On the other hand, high quality OCT *en face* images can be obtained at the same time as the OCTA scanning. The AngioPlex Elite 9000 with 100,000 A-scans/second and a real-time eye tracking system (FastTrac^™^) can record 12 × 12-mm OCTA images and structured *en face* images at the same time. The standard high-resolution OCT *en face* images were used for the measurements. The segmentation boundaries and widths were manually adjusted to the choroidal areas to be analyzed by the embedded software. Only images with signal intensities of 8 or more were used to maintain the reliability of the analyses.

### Measurements of choroidal thickness

The AngioPlex Elite 9000 can record 500 horizontal 12 mm cross sectional scans with the OCTA/*en face* OCT scanning procedures. The subfoveal choroidal thickness (SCT) through the fovea was measured using the caliper tool in the embedded OCT software. The choroidal thickness was defined as the distance between the basal border of the RPE and the chorioscleral junction.

### OCT *en face* images of choroidal vasculature

**Fig 1:**
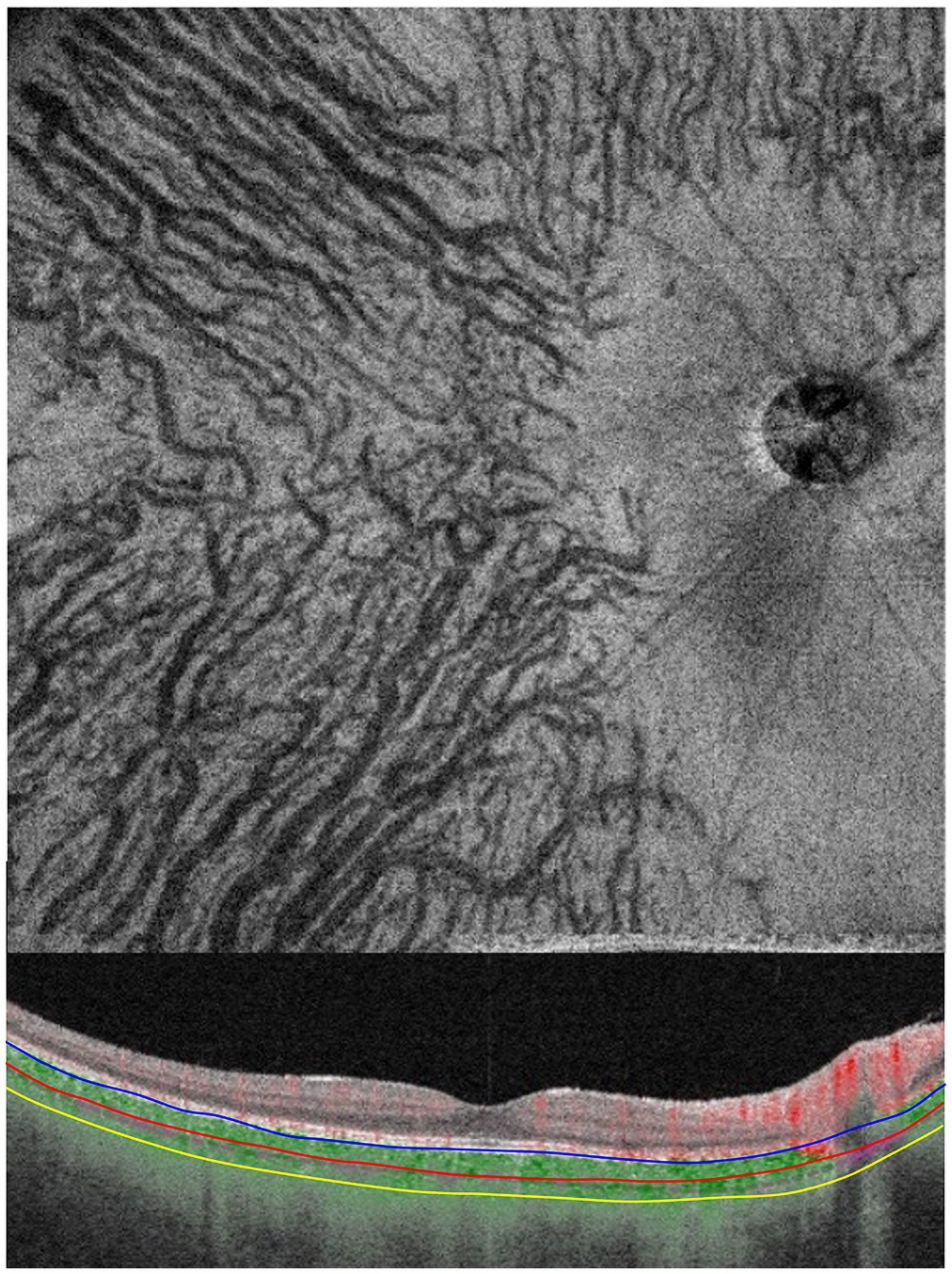
High-quality vascular *en face* image of the choroidal vascular structure at the selected segmentation slab of one-half of the subfoveal choroidal thickness.

The standard *en face* images were flattened at Bruch membrane to quantify the thicknesses of the different structures of the choroid. The segmentation slab selected for the analyses was set at one-half of the subfoveal choroidal thickness with a 30 μm width for the analyses as reported (Fig 1) [28]. After segmentation, the image was cropped to a square and adjusted so that the center of one side of the square was at the center of the temporal edge of the optic disc. This excluded the information of the optic disc from the analyses. When peripapillary atrophy, observed as a whitish area around the optic disc, was present on the temporal side of the optic disc, the area to be analyzed was further cropped to remove the atrophic areas.

### Deep learning

We used two Convolutional Neural Network (CNN) models: the Neural Network Console (NNC; SONY) and the Keras-Tensorflow backend (Google). The VGG16 model is comprised of five blocks with three fully connected layers. Each block includes the convolutional layers followed by a max-pooling layer. A flattening of the output from block 5 resulted in two fully connected layers. In the current study with the Keras model, we adopted and implemented VGG16, a pre-trained CNN architecture that won the ImageNet Large Scale Visual Recognition Challenge (ILSVRC). The Keras model is fine-tuned by transfer learning. The NNC model resembles the VGG16 and was trained from random initialization. We compared the performance of Keras which uses conventional programming to that of NNC which is simpler in function but does not require programming knowledge.

The original *en face* image (550 x 550 pixels) was cropped to remove the optic disc and resized to 224 X 224 pixels. The cropped and resized image was used for both models. The entire 100 *en face* images were randomly divided into 80 for training and 20 for validation. We calculated the correct answer rate during the validation process. For this, we divided it into five groups of 20 eyes each, trained the remaining 80 eyes in each group, and then calculated the correct answer rate for each group by validation with 20 eyes. Thus, each group was trained and validated in the same process for a total of five times as a cross-validation of the method.

The NNC models were trained with a batch size of 20, epochs of 100, and with Adam optimization (learning rate 0.001). The Keras models were trained with a batch size of 10, epochs of 100, and with SGD optimization (learning rate 0.001). The batch size was adjusted for each model to avoid over-training. SGD optimization was used in the Keras model to implement the Heatmap.

### Heatmap with gradient-weighted Class Activation Mapping (Grad-CAM) implemented in Keras

Determining the characteristics of all the convoluted layers that we analyzed with Grad-CAM implemented in Keras were evaluated as heatmaps of the areas that were analyzed by DL [33–35]. The heatmap images were superimposed on the choroidal OCT *en face* vascular images to determine where the DL system was focusing its attention on the choroid. The heatmap images were also exported to ImageJ, and the active region was calculated as a percentage of the cropped image using a threshold value of 128 out of 255 gradations in the heatmap image generated by Grad-CAM. At the time we conducted this study, heatmap display using Grad-CAM was not available for NNC which is why we did not compare heatmap analysis between the two models.

### Statistical analyses

Mann–Whitney U-tests were used to compare the age, choroidal thickness, and active region in the heatmap images between normal and CSC groups. McNemar tests were used to compare the accuracy rate of Keras and NNC models. All *P*-values were two-sided, and *P* values <0.05 were considered statically significant. All statistical analyses were performed with EZR free software (Saitama Medical Center, Jichi Medical University, Saitama, Japan), which is a graphical user interface for R (The R Foundation for Statistical Computing, Vienna, Austria) [36]. More exactly, it is a modified version of R commander designed to add statistical functions that are frequently used in biostatistics.

## Results

**Fig 2:**
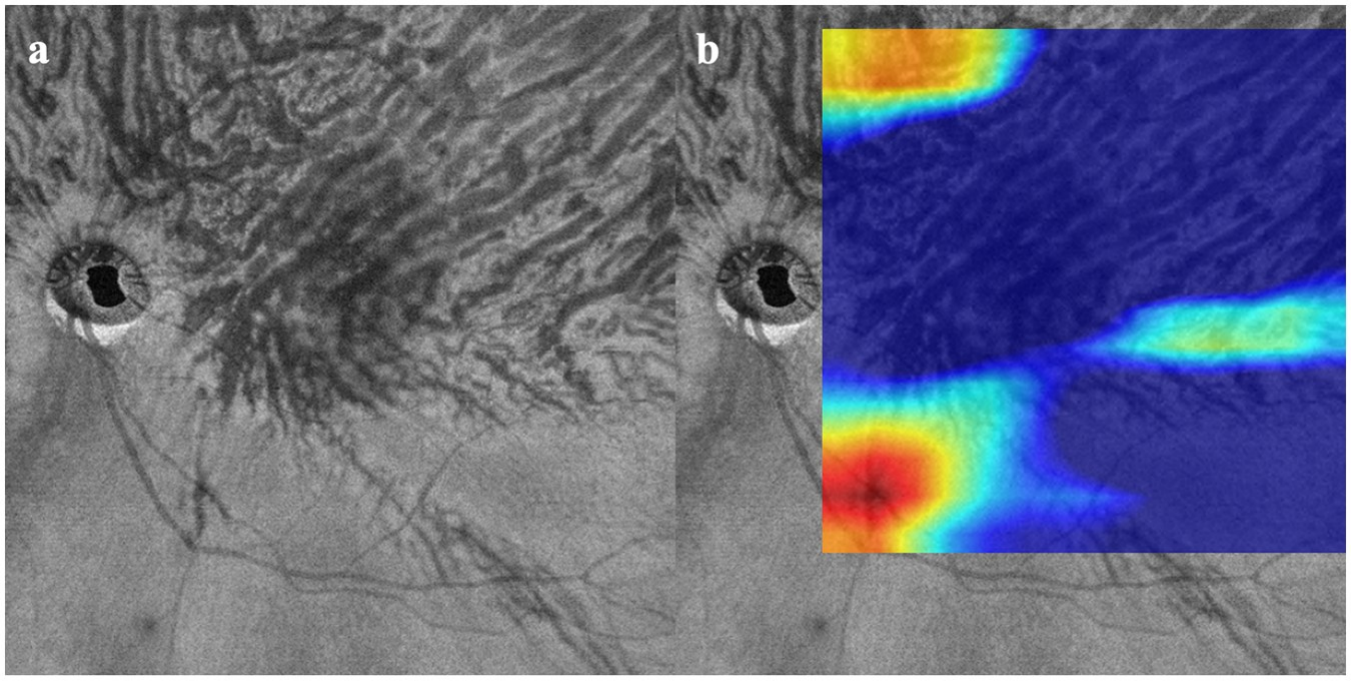
Images from eye with central serous chorioretinopathy (CSC). a. Optical coherence tomographic (OCT) *en face* image of choroidal vasculature. Dilatated choroidal vessels have an asymmetrical pattern flowing to the superior sector. Both the Convolutional Neural Network models of NNC and Keras determined that this image was obtained from an eye with CSC. b. Heatmap superimposed on OCT *en face* image. Deep learning did not focus on the large and dilatated choroidal vessels. The active region was 11.26%.

**Fig 3:**
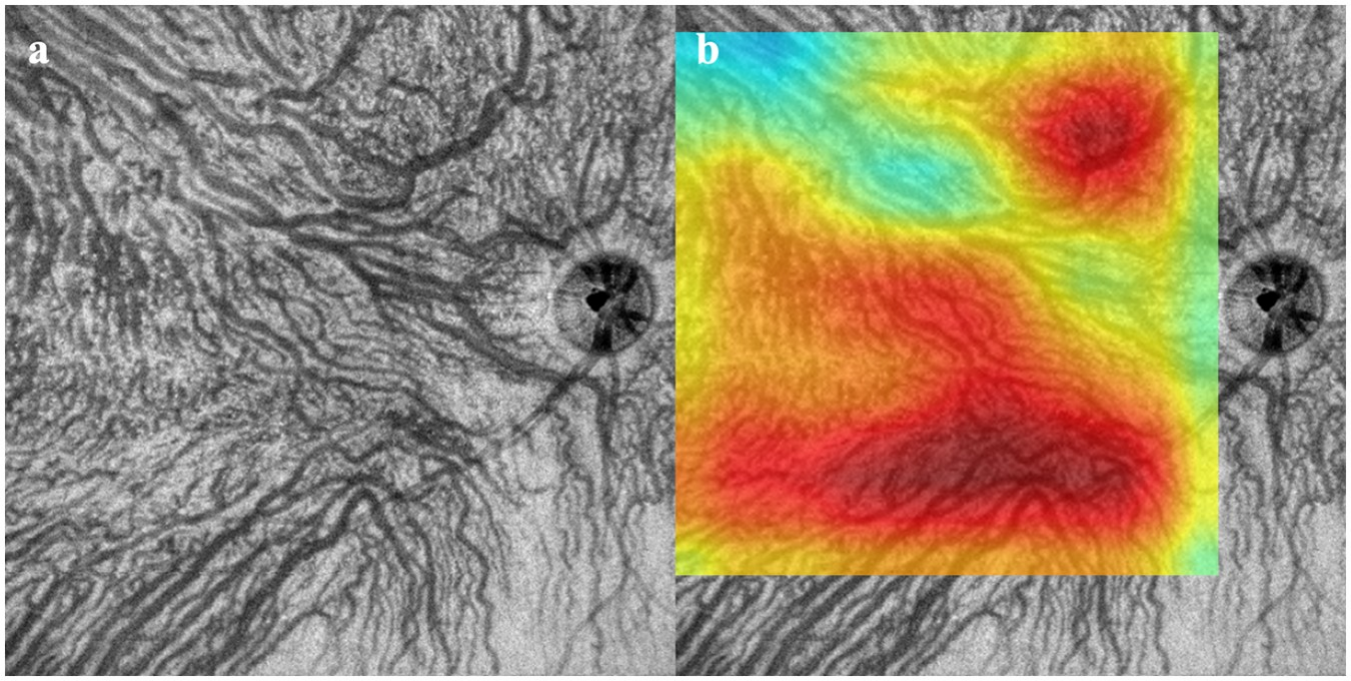
Images from normal eye. a. Choroidal vessels are distributed in a symmetrical pattern from the superior and inferior sectors. Both Convolutional Neural Network models determined that this was obtained from a normal eye. b. Heatmap superimposed OCT *en face* image. Deep learning focused on the uniform choroidal vessels and the area occupied by choroidal vessels in the image. The active region was 92.22%.

**Fig 4:**
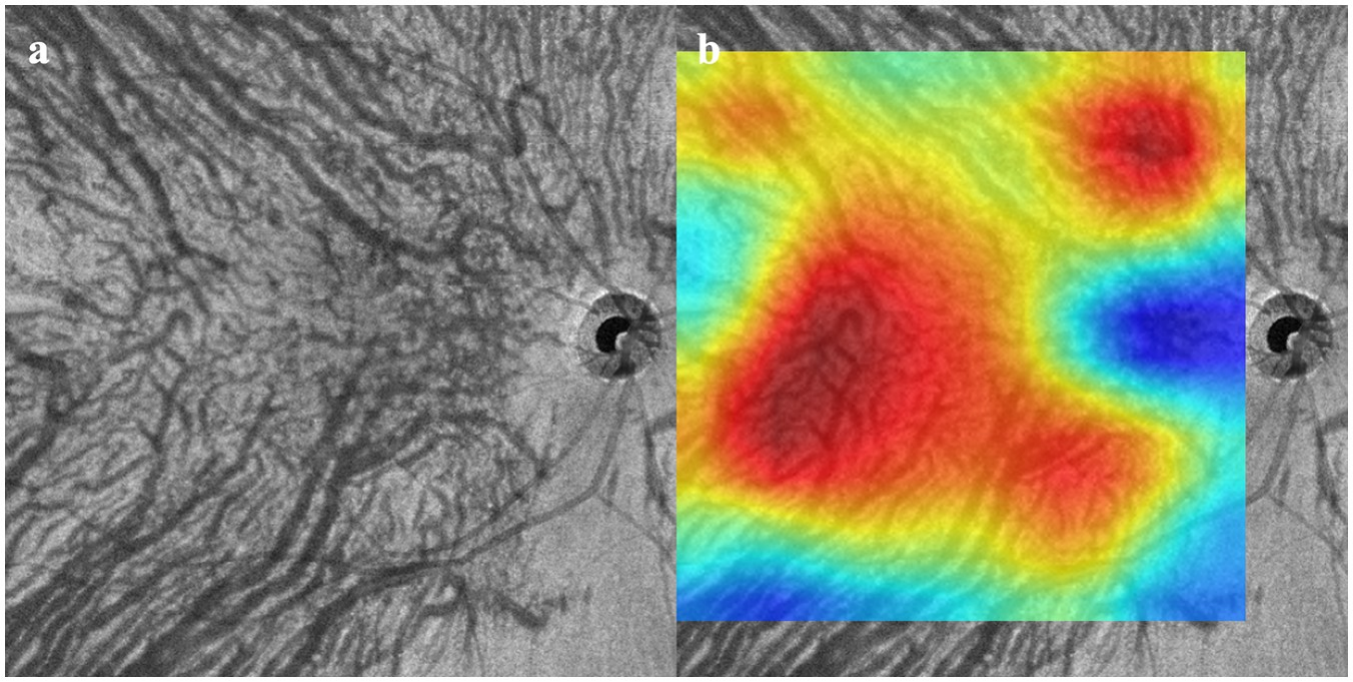
OCTA images from eye with central serous chorioretinopathy (CSC). Some choroidal vessels are dilated, and the vascular density is high. However, the choroidal vessels are distributed in a vertically symmetrical pattern. The NNC model determined that this image was from a CSC eye, but the Keras model judged from a Normal eye. b. Heatmap superimposed on OCT *en face* image. DL focused on the uniformity of the choroidal vessels and the area occupied by the choroidal vessels in the image. The active region is 74.24%.

Fifty-three eyes (26 right, 27 left) of 49 patients with CSC and 47 eyes (23 right, 24 left eyes) of 47 individuals without any retinal and choroidal disorders were studied. The mean age was 51.7 ± 12.7 years in the CSC group which was significantly older than the 37.5 ± 5.4 years in the normal group (*P* <0.01). The subfoveal choroidal thickness (SCT) was 480 ± 92 μm in the CSC group which was significantly thicker than the 292 ± 64 μm in the normal group (*P* <0.01). Representative cases are shown in Figs 2–4.

### Deep learning and heatmap

The mean accuracy rate was 88% for Keras and 95% for NNC. The accuracy rates in each group were 95%, 95%, 95%, 75%, and 80% for Keras and 100%, 95%, 95%, 95%, and 90% for NNC. The differences between the two models were not significant (all *P* >0.1). The results by group and by model for individual eyes are summarized in the supporting information.

The mean active region (± SD) in the heatmap image generated by Grad-CAM was 12.5 ± 23.7% for CSC eyes which was significantly lower than the 79.8 ± 17.3% for normal eyes (*P* <0.01). One case of CSC (Fig 4) had a high value of 74.24%, and this case was incorrectly determined as normal in Keras. If that case was excluded, the mean active region in the CSCs eyes was very low at 3.7 ± 4.2%.

## Discussion

Our results showed that both of our DL models can accurately distinguish normal from CSC eyes by analyzing high-resolution choroidal vascular *en face* OCT images. The DL analyses focused on the uniformity of the choroidal vessels and the area occupied by the choroidal vessels in the images.

Imaging diagnosis using DL in the medical field is rapidly developing and has become common in the fields of radiology and pathology [37–40]. In ophthalmology, it has also been shown to be useful in the diagnosis and staging of various diseases such as diabetic retinopathy [4–6], glaucoma [7–9], age-related macular degeneration [10, 11], and retinopathy of prematurity [12–14]. In CSC, many diagnostic challenges have been investigated using AI including DL. Khalid et al. [18] reported a system that can automatically differentiate normal from CSC or AMD eyes from cross sectional OCT images. Hasssan et al. [19] focused on the retinal thickness to distinguish normal and CSC eyes, and Yoon et al. [20] reported that it was possible to diagnose acute and chronic CSC using cross sectional OCT images. However, they only focused on the retinal tomographic findings. In fact, the cause of CSC was considered to be the choroid. Although the choroid can be evaluated by thickness alone in the tomographic images, it would be better to evaluate the choroidal vascular abnormalities on a wider scale than just a few cross-sectional images. We have also reported that choroidal vascular density has better specificity than choroidal thickness alone for classification of CSC and normal [28]. In the current study, we applied DL methods to evaluate the pattern of the choroidal vessels, the primary cause of CSC. The diagnosis of CSC is usually made by OCT and angiography in addition to fundus findings and cannot be determined by *en face* OCT alone. The results of this study showed that DL can be used to diagnose typical CSC eyes accurately based on the choroidal vascular pattern. A side benefit of DL is that the findings that were previously thought to be ambiguous may be defined with certainty by re-evaluating them from a DL perspective. In addition, it is expected that the use of DL will lead to new findings that have not been noticed even by specialists.

We determined what characteristics were examined by DL of the Keras model using heatmaps with a Grad-CAM implementation. Until now, retinal specialists have observed the choroidal vascular characteristics in OCT *en face* images using choroidal vascular dilation, flow pattern, and density. However, our results showed that DL may have focused on the uniformity of the choroidal vessels and were more evenly distributed in the images of normal eyes than in CSC eyes. It is true that the choroidal vessels are only visible in the upper or lower half of the image in some eyes with CSC. While we were focusing on the asymmetry of the choroidal vessel pattern, we were ignoring the fact that uniformity itself is a diagnostic marker. This unprecedented perspective may be an expected aspect of DL in the future.

Because there was almost no difference in the diagnostic results between the two DL methods, we can reasonably expect that NNC would be easier to use in clinical applications. However, at the time of the study, NNC was not equipped with heatmap analysis, so the diagnostic process could not be read. It appears that the NNC should be used carefully in clinical practice.

There are limitations in this study including its retrospective nature, and it was performed on only a small number of cases. In addition, there was a significant difference in the ages in the normal and CSC groups. This is important because the choroid is thicker, and the density of the vessels is higher in younger individuals. These differences need to be considered especially when the two groups are highly similar. However, this age difference is unlikely to have affected the DL because the results were almost completely classified using the DL. In addition, even though large choroidal vessels existed in the deeper layers, we have evaluated only one-half of the choroidal vascularity in the *en face* OCT images. However, we believe that this method has already been reported and is adequate to assess the medium and large vessels in the choroid with good accuracy [28, 41]. The blood vessels information at the optic disc was excluded from the choroidal blood flow while the artifacts due to large retinal vessels cannot be excluded. Because the luminal area in the retinal blood vessel is much smaller than in the choroidal blood vessel, it is generally believed that there is only a small influence of the retinal vessels. We also had cropped the *en face* images to exclude the influence of the optic disc in our analyses. Thus, these variables have been excluded in the analyses.

In conclusion, DL using 2 CNN models can accurately differentiate normal and CSC eyes from the *en face* OCT images of the choroidal vasculature alone. Previously, the classification mechanism was generally a black box but according to a heatmap analysis using Keras with Grad-CAM implementation, DL does not evaluate the choroidal vessel dilation or flow pattern, it focuses on its uniformity and the area occupied by choroidal vessels in the image. It was interesting to see how these results differed from the focus of retinal specialists. In the future, physicians may not only be receiving assistance in diagnosis of DL, but they may also learn a new perspective from the process of reaching that diagnosis.

## Supporting information

S1 Table (XLSX)

S2 Table (XLSX)
